# Disease Exposure and Antifungal Bacteria on Skin of Invasive Cane Toads, Australia

**DOI:** 10.3201/eid2509.190386

**Published:** 2019-09

**Authors:** Chava L. Weitzman, Mirjam Kaestli, Karen Gibb, Gregory P. Brown, Richard Shine, Keith Christian

**Affiliations:** Virginia Polytechnic Institute and State University, Blacksburg, Virginia, USA (C.L. Weitzman);; Charles Darwin University, Casuarina, Northern Territory, Australia (M. Kaestli, K. Gibb, K. Christian);; University of Sydney, Sydney, New South Wales, Australia (G.P. Brown, R. Shine);; Macquarie University, Sydney (G. P. Brown, R. Shine)

**Keywords:** Australia, cane toad, chytridiomycosis, antifungal, microbiome, Batrachochytrium dendrobatidis, Rhinella marina, invasive species, bacteria, skin, Australia, fungi, amphibians

## Abstract

Cane toads, an invasive species in Australia, are resistant to fungal pathogens affecting frogs worldwide (*Batrachochytrium dendrobatidis*). From toad skin swabs, we detected higher proportions of bacteria with antifungal properties in Queensland, where toad and pathogen distributions overlap, than in other sites. This finding suggests that site-specific pathogen pressures help shape skin microbial communities.

The westward and southward spread of invasive cane toads (*Rhinella marina*) in Australia since their introduction to Queensland in 1935 threatens many native species. In addition to their skin-secreted bufotoxins affecting predators, toads are resistant to the fungal pathogen *Batrachochytrium dendrobatidis* associated with global frog die-offs, but their capacity to spread the pathogen to other frog species remains unclear (*1*,*2*).

As a skin pathogen, *B. dendrobatidis* interacts not only with the host’s immune system, but also with other community members in the skin microbiome (*3*). Many bacteria on frog skin have antifungal properties that can help the host fight *B. dendrobatidis* (*4*), and the presence of bacteria with anti–*B. dendrobatidis* capacity may increase a host’s pathogen resistance. In a previous study about gene expression in experimentally infected cane toads, their strong resistance to *B. dendrobatidis* was not attributed to strong immune function (*1*). Thus, the skin environment, including microbes inhabiting it, alongside an immediate, localized immune response, might play a large role in fighting the pathogen and resisting disease (*1*).

Invading species are predicted to invest in less costly immune defenses while decreasing their investment in costly inflammatory immune responses (*5*). With the assumption that skin bacteria are relatively inexpensive for the host to maintain, we used skin swab samples collected in 2017 to test whether cane toads have increased proportions of putative *B. dendrobatidis*–inhibiting bacteria at the invasion front in Australia, consistent with a previously reported increased investment into low-cost innate immune functions (*6*). Alternatively, we predicted that patterns of *B. dendrobatidis*–inhibitory bacteria on toad skin might depend on the current distribution of, and thus likely exposure to, *B. dendrobatidis*. Our 4 sampling locations (8–18 per site; Appendix Figure 1) comprised 2 sites overlapping the current *B. dendrobatidis* distribution (Queensland) and 2 sites outside the area of threat of chytridiomycosis (Northern Territory and Western Australia). These 4 sites also represent the toad’s westward expansion; Western Australian toads were sampled near the invasion front. Sampling was in accordance with Charles Darwin University Animal Ethics permit A14012.

We compared bacterial amplicon sequence variants (sequences available on FigShare, https://doi.org/10.6084/m9.figshare.7855670) against a database of known anti–*B. dendrobatidis* isolates from the skin of frog species around the world (*4*). We detected 63 bacterial types with previously described anti–*B. dendrobatidis* function in the wild toad samples in our study. The 4 sampling sites differed in the proportion of total sequences and bacterial types represented by putative *B. dendrobatidis*–inhibitory bacteria, and toads from Queensland sites had proportionately more of these sequences and taxa than did toads from other sites (Figure). Some of the *B. dendrobatidis*–inhibitory bacteria were extremely common among our samples (particularly at the Tully site in Queensland [Appendix]).

**Figure:**
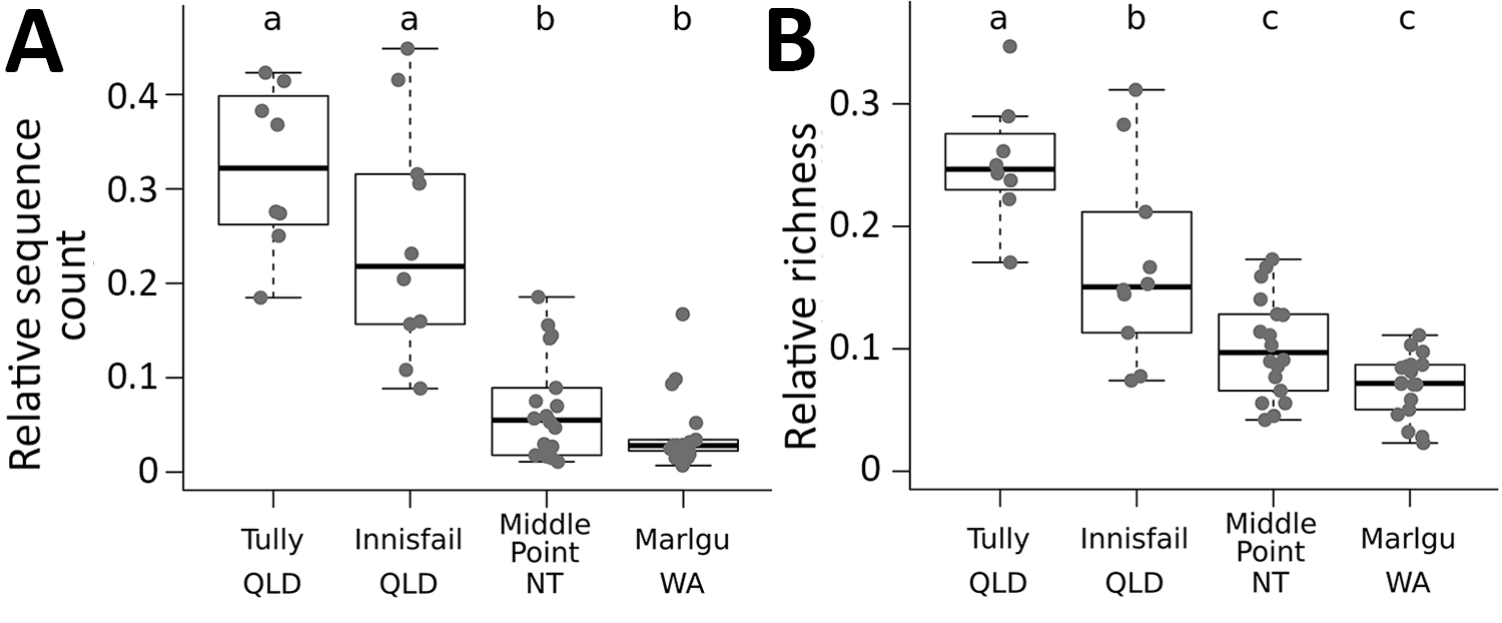
Proportions of sequences (A) and richness (B) represented by *Batrachochytrium dendrobatidis*–inhibitory bacteria detected on the skin of invasive cane toads (*Rhinella marina*) at 4 sites in Australia, 2017. Points indicate values for individual toads. Box plots indicate the median (thick line), interquartile range (box), reasonable range of the data (dashed lines to the whiskers), and outliers. Letters above plots indicate significant differences from Tukey’s post hoc tests with p<0.05. QLD, Queensland; NT, Northern Territory; WA, Western Australia.

Our results indicate that the skin bacterial communities on toads from sites overlapping the *B. dendrobatidis* distribution contain more putative *B. dendrobatidis*–inhibitory bacteria than is the case for toads from sites not yet invaded by the pathogen. Rather than following predictions regarding immunocompetence at the invasion front, this pattern suggests that *B. dendrobatidis*–inhibitory bacteria are selected for where the disease is present, in concordance with the adaptive microbiome hypothesis presented by Jin Song et al. (*7*). Outside of the *B. dendrobatidis* range, selection for anti–*B. dendrobatidis* microbes is relaxed; inhibitory microbes represent less of the community, and some disappear.

In cane toads, juvenile life stages succumb to *B. dendrobatidis*, although they have higher survival rates and better ability to clear an infection than other amphibians (e.g., *1*). The prevalence of bacteria with *B. dendrobatidis*–inhibitory capacity on adult cane toads in Queensland suggests that the skin microbiome might confer some of the resistance to disease in this host species. Although amphibian skin microbiome communities change across ontogeny, host species is a strong predictor of skin communities across life stages (*8*). Thus, the communities found on these adult toads may predict those found on juvenile toads.

Our results could be affected by larger differences in bacterial community composition that can occur among sampling sites in cane toads (*9*) and other frog species (*8*). These differences could be due to diverse environmental microbiota supported by climatic and other abiotic and biotic conditions that change across the landscape.

The detection of *B. dendrobatidis*–inhibitory microbes at *B. dendrobatidis*–naive sites might be misleading. The presence of a functional gene does not necessarily imply gene activity (*10*). Thus, the approach of ascribing *B. dendrobatidis*–inhibitory roles based on presence might be too simplistic in the absence of direct evidence of *B. dendrobatidis*–inhibitory action, which was outside the scope of this study. Some of these bacteria may be commonly found on cane toad skin, regardless of an active function to inhibit *B. dendrobatidis*. Nonetheless, the higher frequency of these bacteria in *B. dendrobatidis*–exposed locations suggests that the microbiome on the skin of cane toads is shaped, at least partly, by natural selection in response to geographic variation in the degree of threat posed by specific diseases.

Appendix Additional methods and results for study of disease exposure and antifungal bacteria on skin of invasive cane toads, Australia.
