# Supplementary material for: Disease Exposure and Antifungal Bacteria on Skin of Invasive Cane Toads, Australia
Source: Emerg Infect Dis. 2019 Sep;25(9):1770–1. doi: 10.3201/eid2509.190386 (PMC6711215; doi:10.3201/eid2509.190386)
Supplement: Appendix — Additional methods and results for study of disease exposure and antifungal bacteria on skin of invasive cane toads, Australia. [file 19-0386-Techapp-s1.pdf]

Article DOI: https://doi.org/10.3201/eid2509.190386

Disease Exposure and Antifungal Bacteria on Skin of Invasive Cane Toads, Australia

Appendix

Sample Collection and Microbiome Sequencing

In September 2017, cane toads (*Rhinella marina*) were swabbed for skin-associated bacteria at 4 sampling sites in Australia: Tully, Queensland (n = 8); Innisfail, Queensland (n = 10); Middle Point, Northern Territory (n = 18); and Marlgu Billabong, Western Australia (n = 18), near the cane toad invasion front (Appendix Figure 1). Free-ranging toads were caught by hand or net and rinsed with 100 mL 0.45 μm high-purity water (*1*,*2*) before collecting swab samples with a sterile synthetic swab (Transwabs, Cat # MW167S; Medical Wire and Equipment Company, https://www.mwe.co.uk). Swabbing included 30 strokes around the entire toad body, excluding the cloaca and head, following protocols in Christian et al. (*3*). Toad handlers wore new gloves for each swabbed animal. Swab samples were placed on ice in the field before frozen at –20°C.

DNA was extracted from swab samples using the Norgen's Swab Collection & DNA Preservation Kit (Cat # 45681, Norgen Biotek Corp., https://norgenbiotek.com) following the protocol. Extracted DNA was quantified, and we sent 200 ng of dried DNA to ACE Sequencing Service at the Australian Centre for Ecogenomics (University of Queensland) for Illumina MiSeq sequencing (Illumina, https://www.illumina.com).

Sequencing targeted the V4 region of small subunit rDNA using F515/R806 primers (*4*). Sequencing was conducted on multiplexed samples, which included additional toad swab samples and 2 negative controls based on DNA extracted from clean swabs. Sequences were processed to amplicon sequence variants (ASVs) by ACE with the following pipeline. The software Trimmomatic was used for sequence quality trimming removing poor quality sequences with a sliding window of 4 bases and an average base quality >15. All reads were hard trimmed to 250 bases, and any shorter reads were excluded. Reads were processed to ASVs using the

QIIME2 workflow with default parameters and the DADA2 algorithm (*5*,*6*). Taxonomic assignment of ASVs was through BLAST+ using the reference database SILVA (https://www.arb-silva.de). Only ASVs identified as Bacteria were kept. Bacterial ASVs and associated metadata are available on FigShare: https://doi.org/10.6084/m9.figshare.7855670.

After inspecting rarefaction curves, we subsampled data to the lowest sequence count (2,744) per toad sample, corresponding with 41 to 311 bacterial types per sample.

We used QIIME2 to compare feature sequences against a database of isolates whose fungal inhibitory capacity have previously been tested against the fungal pathogen, *Batrachochytrium dendrobatidis* (*7*). The Antifungal Isolates Database contains information on the amphibian host species the bacterium was isolated from, geographic location where the host was caught and sampled, fungal-inhibition results, and bacterial taxonomy from UClust (*8*) and RDP (*9*). The database contains information on >1,100 bacterial isolates with capacity for *B. dendrobatidis*–inhibition.

We used the "cluster-features-closed-reference" option to identify feature sequences in our dataset that were at least 99% similar to sequences in the Woodhams et al. database (*7*). Resultant data were then separated into *B. dendrobatidis*–inhibitory and *B. dendrobatidis*–enhancing bacteria, and we determined sequence count and richness per sample. Because negative controls had sequence counts too low to be retained after rarefaction (<600 sequences each), we compared negative control data against the Woodhams et al. database separately for qualitative purposes. Lastly, we identified which *B. dendrobatidis*–inhibitory bacterial types were present in the majority of samples from each sampling site (often referred to as "core" microbiome; 87.5% prevalence cutoff).

Statistical Analyses

We used Kruskal-Wallis tests to compare richness and Shannon diversity of the rarefied microbiome communities to detect inherent differences among the sites. Similar to analyses in Varela et al. (*10*), we compared the proportion of total sequences or richness represented by *B. dendrobatidis*–inhibitory bacteria using generalized linear models. Inhibitory data were analyzed using a quasibinomial distribution, with richness proportions weighted for total richness. *B. dendrobatidis*–enhancing sequence count data were analyzed using a quasipoisson distribution,

because the values were small (maximum = 5.3% of sequences) and showed overdispersion. Tukey's post-hoc tests were used for pairwise site comparisons using the glht function in the multcomp R package (*11*). All analyses were conducted in R v. 3.4.4 (*12*).

Results

We detected 63 *B. dendrobatidis*–inhibitory bacterial types in our samples, with 4 to 23 per sample. The negative controls had 2 *B. dendrobatidis*–inhibitory bacterial types, 1 of which was not present in our wild toad samples. The second, *Stenotrophomonas* sp. (Phylum: Proteobacteria), was present in wild toad samples and was a "core" OTU in Tully samples (Appendix Table). Known *B. dendrobatidis*–inhibitory bacteria represented up to 45% of the total sequences in a sample. Most of the *B. dendrobatidis*–inhibitory bacteria were originally isolated from amphibians in Latin America, with additional bacteria from the United States and Madagascar. Nine were isolated from *Litoria* spp. of frogs from Queensland, Australia.

From Kruskal-Wallis tests, we did not find significant differences in richness (χ^2 = 6.2061, df = 3, p = 0.102) or Shannon's diversity index (χ^2 = 5.242, df = 3, p = 0.1549) among our sites.

Generalized linear models found significant differences among sites in relative *B. dendrobatidis*–inhibitory sequence count and richness: proportion inhibitory sequences, $F_{(3,50)}$ = 35.34, $p<0.0001$; proportion inhibitory richness, $F_{(3,50)}$ = 26.24, $p<0.0001$. From Tukey's post hoc tests for pairwise comparisons between sites, toads from Queensland sites had a greater proportion of sequences and bacterial types represented by *B. dendrobatidis*–inhibitory bacteria (Figure).

Of the 63 *B. dendrobatidis*–inhibitory bacteria in our samples, 14 were in the "core" microbial community (microbes with 87.5%+ prevalence) of one or more geographic sampling sites. The Tully site in Queensland had 13 known *B. dendrobatidis*–inhibitory bacteria in its core community, which was likely influenced both by the site's small sample size of 8 and the presence of chytridiomycosis in the region. The other sites had only 2 to 5 *B. dendrobatidis*–inhibitory bacterial types in their core microbiota. Three *B. dendrobatidis*–inhibitory bacteria were commonly found on toads from all 4 sites sampled in our study (in $\geq$46 of our 54 samples): *Sphingobacterium multivorum* (Phylum Bacteroidetes), originally isolated from a Waterfall Frog

in Queensland; a *Microbacteriaceae* (Phylum Actinobacteria), originally isolated from an American Bullfrog in Colorado, USA; and an *Enterobacteriaceae* (Phylum Proteobacteria), originally isolated from a *Bufo typhonius* toad in Panama. The Appendix Table contains a list of the bacteria found in at least 87.5% of the samples per site.

Our cane toad samples had only 2 *B. dendrobatidis*–enhancing bacteria from the Woodhams et al. 2015 database (*7*), both originally isolated from Panamanian frogs: *Acinetobacter rhizophaerae* (Phylum Proteobacteria) and *Microbacterium* sp. (Phylum Actinobacteria). *B. dendrobatidis*–enhancing bacteria represented up to ≈5% of the total sequences and richness in a sample. From generalized linear models, sites were found to differ in proportion of *B. dendrobatidis*–enhancing sequences ($F_{(3,50)} = 6.08$, p = 0.001). Some pairwise differences were detected between sites, with toads from the Northern Territory having greater abundance of *B. dendrobatidis*–enhancing bacteria than toads from both Queensland sites (Appendix Figure 2). The Woodhams et al. database (*7*) contains only 58 known *B. dendrobatidis*–enhancing isolates, none of which were isolated from Australian species. The database was compiled to identify bacteria that may aid in the fight against the pandemic frog chytridiomycosis, and as such, is skewed toward antifungal, and not fungal-enhancing bacteria. Nevertheless, in the face of this disease, the presence of *B. dendrobatidis*–enhancing bacteria representing nearly 5% of the bacteria on a disease-resistant, invasive cane toad's skin could be an important consideration when seeking to understand chytridiomycosis on native Australian frogs.

References

1. Culp CE, Falkinham JO III, Belden LK. Identification of the natural bacterial microflora on the skin of eastern newts, bullfrog tadpoles and redback salamanders. Herpetologica. 2007;63:66–71. https://doi.org/10.1655/0018-0831(2007)63[66:IOTNBM]2.0.CO;2

2. Lauer A, Simon MA, Banning JL, André E, Duncan K, Harris RN. Common cutaneous bacteria from the eastern red-backed salamander can inhibit pathogenic fungi. Copeia. 2007;2007:630–40. https://doi.org/10.1643/0045-8511(2007)2007[630:CCBFTE]2.0.CO;2

3. Christian K, Weitzman C, Rose A, Kaestli M, Gibb K. Ecological patterns in the skin microbiota of frogs from tropical Australia. Ecol Evol. 2018;8:10510–9. PubMed https://doi.org/10.1002/ece3.4518

4. Caporaso JG, Lauber CL, Walters WA, Berg-Lyons D, Lozupone CA, Turnbaugh PJ, et al. Global patterns of 16S rRNA diversity at a depth of millions of sequences per sample. Proc Natl Acad Sci U S A. 2011;108(Suppl 1):4516–22. PubMed https://doi.org/10.1073/pnas.1000080107

5. Caporaso JG, Kuczynski J, Stombaugh J, Bittinger K, Bushman FD, Costello EK, et al. QIIME allows analysis of high-throughput community sequencing data. Nat Methods. 2010;7:335–6. PubMed https://doi.org/10.1038/nmeth.f.303

6. Callahan BJ, McMurdie PJ, Rosen MJ, Han AW, Johnson AJA, Holmes SP. DADA2: High-resolution sample inference from Illumina amplicon data. Nat Methods. 2016;13:581–3. PubMed https://doi.org/10.1038/nmeth.3869

7. Woodhams DC, Alford RA, Antwis RE, Archer H, Becker MH, Belden LK, et al. Antifungal isolates database of amphibian skin–associated bacteria and function against emerging fungal pathogens. Ecology. 2015;96:595–595. https://doi.org/10.1890/14-1837.1

8. Edgar RC. Search and clustering orders of magnitude faster than BLAST. Bioinformatics. 2010;26:2460–1. PubMed https://doi.org/10.1093/bioinformatics/btq461

9. Wang Q, Garrity GM, Tiedje JM, Cole JR. Naive Bayesian classifier for rapid assignment of rRNA sequences into the new bacterial taxonomy. Appl Environ Microbiol. 2007;73:5261–7. PubMed https://doi.org/10.1128/AEM.00062-07

10. Varela BJ, Lesbarrères D, Ibáñez R, Green DM. Environmental and host effects on skin bacterial community composition in Panamanian frogs. Front Microbiol. 2018;9:298. PubMed https://doi.org/10.3389/fmicb.2018.00298

11. Hothorn T, Bretz F, Westfall P. Simultaneous inference in general parametric models. Biom J. 2008;50:346–63. PubMed https://doi.org/10.1002/bimj.200810425

12. R Core Team. R: a language and environment for statistical computing. Vienna (Austria): R Foundation for Statistical Computing; 2014 [cited 2019 Jun 21]. http://www.R-project.org.

Appendix Table. Core *Batrachochytrium dendrobatidis*–inhibitory bacteria on wild cane toads by sampling site, Australia, 2017*

SampleID	TULLY, n = 8	INN, n = 10	NT, n = 18	WA, n = 18	Region	Uclust_taxonomy
Atelopuslimosus-inhibitory_28	8	9			Panama	p__Proteobacteria; c__Gammaproteobacteria; o__Pseudomonadales; f__Moraxellaceae; g__Acinetobacter; s__
Atelopuslimosus-inhibitory_64	7				Panama	p__Bacteroidetes; c__Flavobacteriia; o__Flavobacteriales; f__[Weeksellaceae]; g__Chryseobacterium; s__
Bufotyphonius-inhibitory_9	7	9		16	Panama	p__Proteobacteria; c__Gammaproteobacteria; o__Enterobacteriales; f__Enterobacteriaceae
Craugastorcrassidigitus-inhibitory_109	8	9			Panama	p__Proteobacteria; c__Betaproteobacteria; o__Burkholderiales; f__Comamonadaceae; g__Comamonas; s__
Craugastorcrassidigitus-inhibitory_121	8	9			Panama	p__Proteobacteria; c__Gammaproteobacteria; o__Pseudomonadales; f__Pseudomonadaceae; g__Pseudomonas; s__
Craugastorcrassidigitus-inhibitory_165	7			17	Panama	p__Bacteroidetes; c__Flavobacteriia; o__Flavobacteriales; f__[Weeksellaceae]; g__Wautersiella; s__
Dendrobatesauratus-inhibitory_5	8				Panama	p__Proteobacteria; c__Gammaproteobacteria; o__Xanthomonadales; f__Xanthomonadaceae; g__Stenotrophomonas; s__
Espadaranaprosoblepon-inhibitory_40	7				Panama	p__Proteobacteria; c__Gammaproteobacteria; o__Pseudomonadales; f__Pseudomonadaceae; g__Pseudomonas; s__
Lithobatescatesbeianus-inhibitory_37	7		16	16	Colorado	p__Actinobacteria; c__Actinobacteria; o__Actinomycetales; f__Microbacteriaceae; g__; s__
Litorianannotis-inhibitory_24	8		16	16	Queensland	p__Bacteroidetes; c__Sphingobacteriia; o__Sphingobacteriales; f__Sphingobacteriaceae; g__Sphingobacterium; s__multivorum
Litorianannotis-inhibitory_52†	7				Queensland	p__Proteobacteria; c__Gammaproteobacteria; o__Xanthomonadales; f__Xanthomonadaceae; g__Stenotrophomonas; s__
Litoriarheocola-inhibitory_25	8				Queensland	p__Proteobacteria; c__Alphaproteobacteria; o__Sphingomonadales; f__Sphingomonadaceae; g__Novosphingobium; s__
Smiliscasordida-inhibitory_34	7				Panama	p__Proteobacteria; c__Gammaproteobacteria; o__Pseudomonadales; f__Pseudomonadaceae; g__Pseudomonas
Strabomantisbufoniformis-inhibitory_11				16	Panama	p__Proteobacteria; c__Gammaproteobacteria; o__Aeromonadales; f__Aeromonadaceae; g__; s__

SampleID	TULLY, n = 8	INN, n = 10	NT, n = 18	WA, n = 18	Region	Uclust_taxonomy
Total *B. dendrobatidis*–inhibitory in core	13	4	2	5		

*SampleID denotes the isolate name from the Antifungal Isolates Database. SampleID includes the frog species from which the bacterium was originally isolated. Region and Uclust_taxonomy data are from the Woodhams et al. (*7*) metadata, indicating where in the world the bacterium was isolated for fungal-inhibition challenge experiments. Toad data are separated by sampling site: INN, Innisfail, QLD; TULLY, Tully, QLD; NT, Middle Point, NT; WA, Marlgu Billabong, WA. Values indicate the number of samples with the bacterial type, where the bacterium was part of the "core" community (i.e., blank spaces do not equal zeros).
†Bacterium found in negative controls.

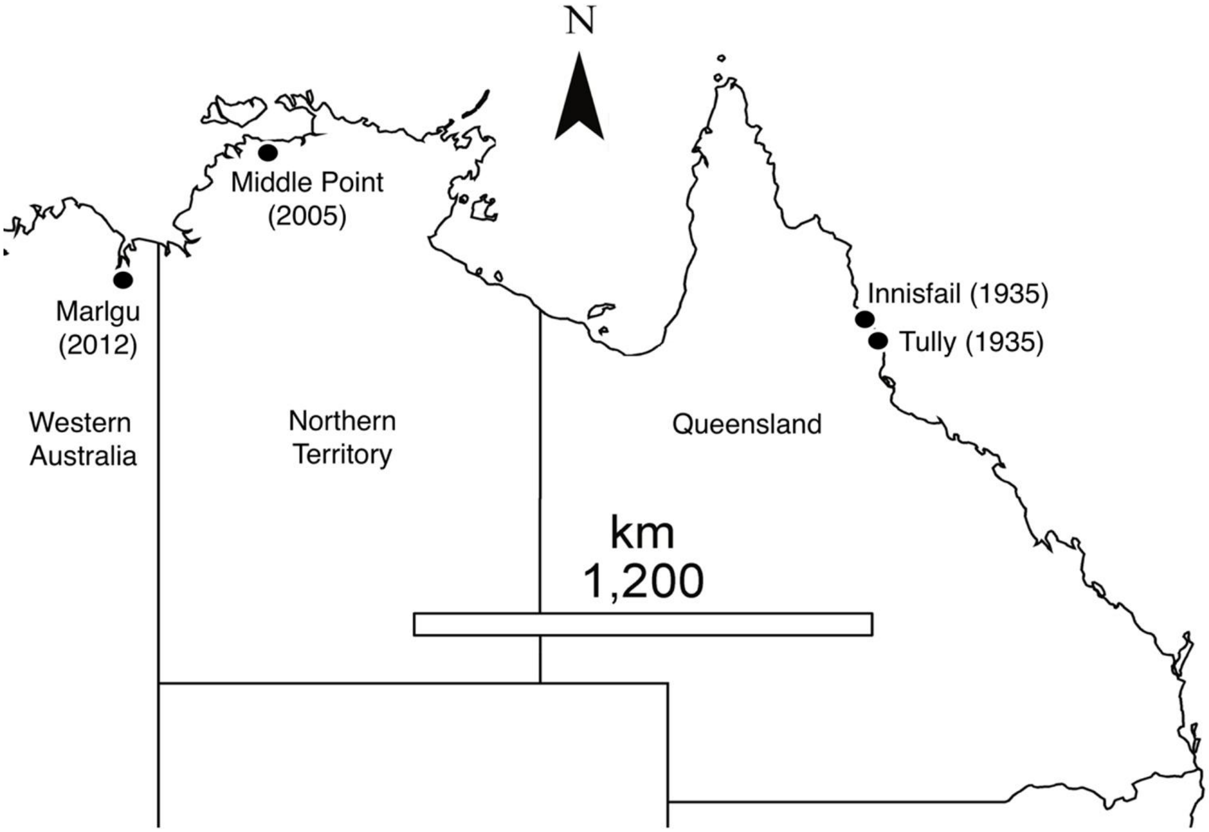

Appendix Figure 1. Four toad sampling sites in northern Australia with years of invasive toad arrival to the sites.

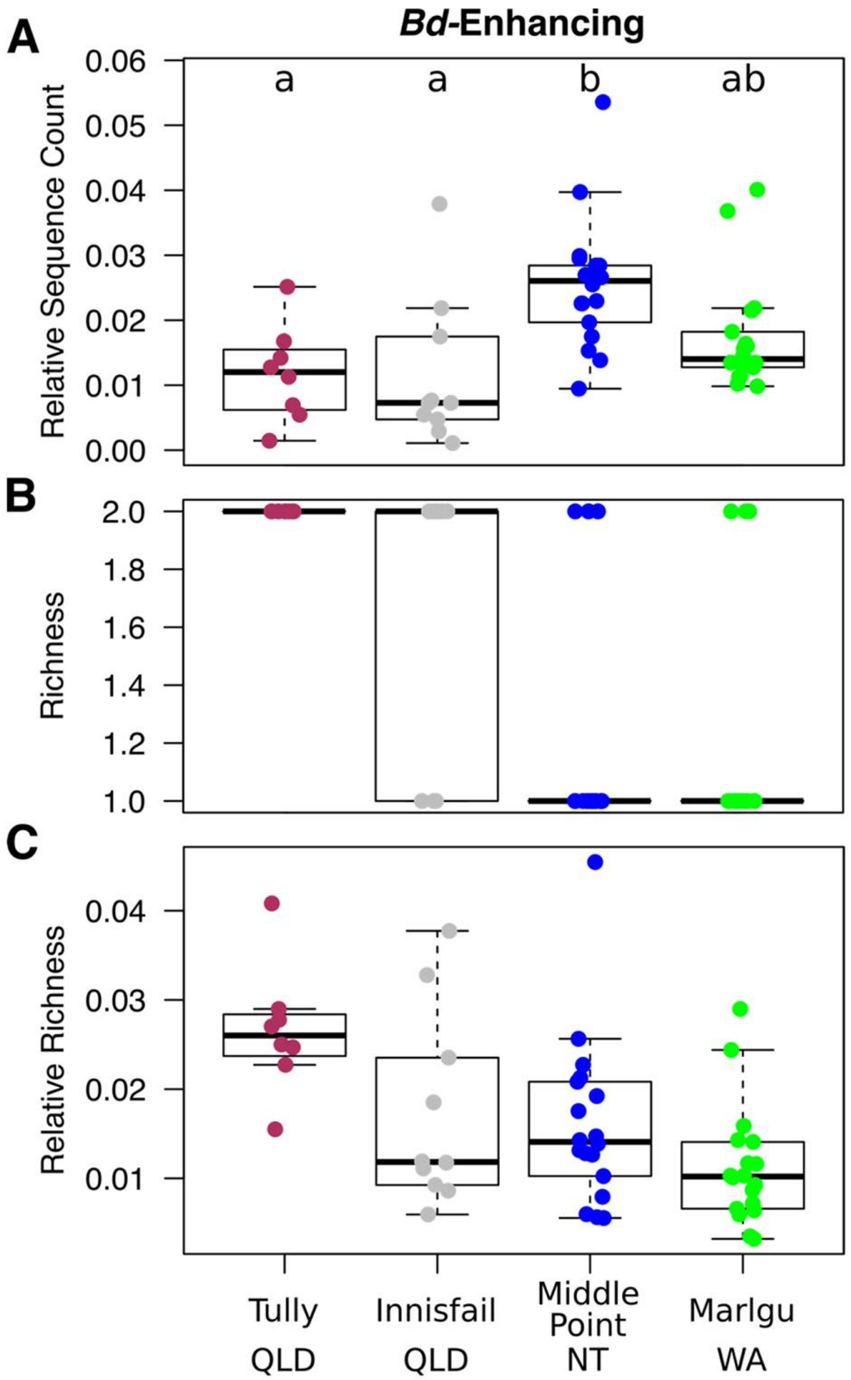

Appendix Figure 2. Boxplots of proportion of sequences (A), total richness (B), and proportion of richness (C) represented by *Batrachochytrium*–enhancing bacteria. Points indicate values for individual toads. Boxplots indicate the median (thick line), interquartile range (the box), reasonable range of the data (dashed lines to the whiskers), and outliers. Letters above plots indicate significant differences from Tukey’s post hoc tests with $p<0.05$. *Bd*, .*Batrachochytrium dendrobatidis;* NT, Northern Territory; QLD, Queensland; WA, Western Australia.
